# Somatic feather follicle cell culture of the *gallus domesticus* species for creating a wild bird genetic resource bank

**DOI:** 10.1590/1984-3143-AR2020-0044

**Published:** 2020-09-18

**Authors:** Camila Alampe Cardoso, Lina Castelo Branco Motta, Vanessa Cristina de Oliveira, Daniele dos Santos Martins

**Affiliations:** 1 Faculdade de Zootecnia e Engenharia de Alimentos, Universidade de São Paulo, Pirassununga, SP, Brasil; 2 Hospital Veterinário, Universidade de Trás-os-Montes e Alto Douro, Vila Real, Portugal

**Keywords:** feather follicle, somatic cells, birds

## Abstract

The creation of a genetic resource bank of avian species aims to prevent the decline and fragmentation of wild bird populations, which in turn lead to the loss of genetic diversity and, in more serious cases, the extinction of the most threatened species. In order for the collected genetic material to be stored in a bank and useful when necessary, it is essential to improve the technique ensuring its effectiveness. Thus, our study used feather follicle cells from the *domestic gallus* species to standardize the technique of cell culture and subsequent cryopreservation. This study aimed to establish a protocol, *in vitro*, of isolation and primary culture of somatic cells derived from the feather follicle, with the purpose of establishing a cell lineage, and evaluate its viability for the biobank formation. Developing feathers of *gallus domesticus* were collected at 12, 21 and 34 days of age. The feathers were morphologically analyzed and then we selected the region of the calamus due to the presence of pulp for cell culture and cryopreservation. The results showed that it is possible to find cells with distinct morphology; cells in elliptical shape with central nucleus also in elliptical shape, cells with shape and round nucleus, cells compatible with the fibers of the barbules, cell agglomerates and cells adhered to the bottom of the plate with fibroblastatoid shape. After 24 hours of culture there was the presence of primary culture with 80% of confluence and after cryopreservation the average viability after freezing was 68.8%, with cellular morphologies being maintained. Therefore, we proved the isolation of somatic cells from the follicle of bird’s feathers, suggesting that this is a source of great value, viable and effective for obtaining biological material for the elaboration of a biobank.

## Introduction

It is well known that Earth's biodiversity is being degraded by several factors, such as urbanization, agribusiness, exploration of raw materials, illegal trade in wild animals, pollution, emerging diseases, invasive exotic animals and plants, and climate changes. (Wildt et al., 2010). These factors endanger the presence of thousands of animal species, especially species in or close to be in danger and genetically diverse, due to natural selection promoted by changes in the environment in which they are inserted. Once a genetic resource has disappeared, it cannot be recovered (Clarke, 2009).

Conservation in situ, ideal as it seems still insufficient, due to the large number of the human population on earth, natural resources are increasingly needed and this ends up making impossible to conserve endangered populations just by preserving their habitat (Monfort, 2014).

Ex situ in vivo conservation, for example zoos, are limited to their restricted spaces, which makes it difficult to meet the demographic and genetic objectives in their conservation programs, thus ex situ conservation in vitro has been gaining prominence and increasingly more studies and conservation projects see using biomaterials of wild species (Wildt et al., 2012).

Some recent examples worldwide that aim to work with biobanks of endangered wild species is the Frozen Ark Consortium that work with cell culture including somatic cells. The BRB of Southern Africa’s Wildlife, the Alfred Brehm (Cryo-Brehm) work with stem cells and the Cell bank for all fauna in danger of extinction in Spain, directed by Dr. Trinidad León Quinto. Both work with different species of wild animals and use different biological samples such as blood, hair, tissue sample, eggshell, gametes among other.

Given these data, it becomes clear the attention that we must give to Brazilian avian species. The use of feather follicle cells is an excellent path due to the ease of obtaining somatic cells from avian species (Ryder, 2002). It is a method of great value, because these cells can provide information about genetic variations, phylogeny, paternity, gene flow, genetic selection, mutations, among others (Xi et al., 2003). This material can also be used for innovative therapies in rehabilitation centers or in stem cell regenerative medicine (Ambrósio et al., 2019) and, ultimately, for the somatic cell nuclear transfer cloning process (Trounson et al., 1998).

The wild fauna biobanks have unique characteristics and/or unique constituents (Comizzoli, 2017). The conservation of genetic resources is an excellent option to reduce the continuous loss of animals due to environmental degradation.The use of non-invasive or minimally invasive methods represents an advantageous solution for the conservation of genetic diversity (Xi et al., 2003). For this reason, our investigation highlights the standardization protocol for primary in vitro culture of feather follicular cells. Our results allowed the cryopreservation of a viable cell line for future research.

## Material and methods

### Animals

All study procedures were performed in accordance with the *Guide for the Care and Use of Laboratory Animals of the National Institutes of Health.* The protocol were performed in accordance with Law 11.794 of October 8, 2008, Decree 6899 of July 15, 2009, as well as, with the rules issued by the National Council for Control of Animal Experimentation (CONCEA), and was approved by the Ethic Committee on Animal Use of the School of Veterinary Medicine and Animal Science (University of Sao Paulo), Brazil (protocol number 6378040618).

## Experimental design

To perform the standardization of the technique we used the species *Gallus domesticus*. The collection was carried out in the aviary of the Faculty of Animal Sciences and Food Engineering of the University of São Paulo, Pirassununga Campus.

The study was performed in the Laboratory of the Innovative Therapies Development Group (GDTI) and in the Laboratory of Immunohistochemistry and Experimental Physiology (LIFE), both belonging to the Department of Veterinary Medicine of the Faculty of Animal Sciences and Food Engineering of the University of São Paulo, Pirassununga campus.

Samples from 15 unrelated birds at different ages were used and divided equally into three groups: 12 days, 21 days, and 34 days, without distinction of gender. Each animal had 4 feathers collected: 2 taken from the primary remige and 2 from secondary remige, on each side, totaling 60 samples.

Tissue antisepsis was performed close to the pen collection site with a solution 1% chlorhexidine and 70% EtOH. The method of collecting the material was made by pulling out the feathers. The removed feathers were stored in Falcon type tubes containing 8 mL of PBS solution. Then, the material was transported in a thermal box, kept at approximately 4 °C.

## Isolation and cell culture

**Figure 1:**
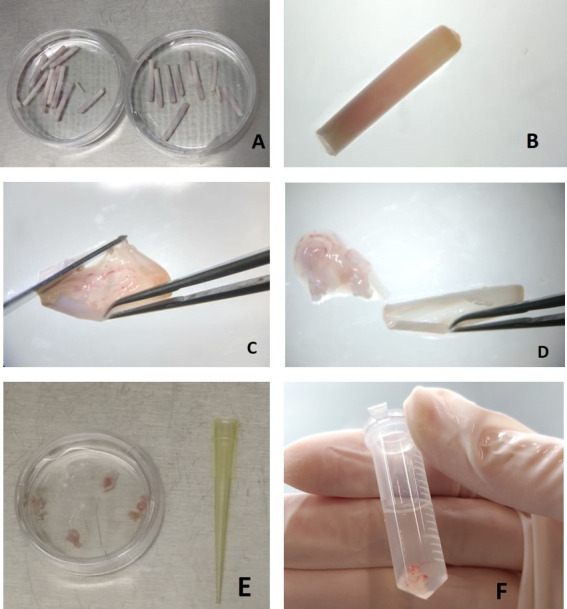
A) Washing of samples in PBS medium containing 2% of penicillin/streptomycin (Sigma-Aldrich®) and 1% amphotericin B, B) Chalamus, C) Longitudinal section of wall of the calamus with the aid of a scalpel and a Drop ship clamp, D) Pulp separated from the calamus wall, E) Washing with PBS solution and F) Eppendorf containing Dulbecco Modified Eagle (Gibco®) and pulp for post-enzymatic digestion neutralization.

For the pulp isolation the calamus was sectioned and the pulp adhered to the wall of the calamus. The structures were carefully detached and completely separating. The pulp was mechanically digested and fragmented into smaller dimensions and then enzymatic digestion was performed. After neutralization, the material was centrifuged and sufficient pellets were obtained to remove and suspend the medium (Figure 1).

The cultivation protocol was adapted from Xi et al. (2003). The samples were washed in PBS, 2% penicillin/streptomycin (Sigma-Aldrich®) and 1% amphotericin B. With the aid of a stereomicroscopic LABOMED 4Z model, a cross section of the calamus region containing the feather pulp was made and then a longitudinal section of the calamus wall was made to remove the internal contents.

Mechanical digestion was performed, followed by enzymatic digestion with 0.1% collagenase IV (Sigma®) for 1 hour it was incubate in a humidified atmosphere 80% and 5% CO2 at 38.5 °C. Until cell suspension was obtained, and then inactivated with culture medium containing Dulbecco-Modified Eagle Media (Gibco®), 10% bovine fetal serum (FBS, Hyclone, Logan, Utah, USA), 1% penicillin/streptomycin (Sigma-Aldrich®), 1% gentamicin, 1% amphotericin B and 1% non-essential amino acid (cat. # M-7145) (100X solution).

The content was centrifuged and the supernatant discarded. The precipitated cells resuspended in the culture medium and 120 µL were inoculated onto a 1-well (growth area 0.33 cm^2^) with 180 µL of culture medium and incubate in a humidified atmosphere 80% and 5% CO2 at 38.5 °C

After 24 hours, the medium was removed and enzymatic disaggregation 0.25% tryple was added (Tryple Express Enzyme- Gibco™). The cell remained in the incubator for 7 minutes at 38.5 °C and 5% CO2 atmosphere. Counting cell was performed in Neubauer Chamber using 10 µL of suspension cell.

For analyse the colony formation capacity the cell was replating using 5x10^4^ cells each well with culture medium and incubate in a humidified atmosphere 80% and 5% CO2 at 38.5 °C for

## Cryopreservation

The cryopreservation occurred in the first passage (P1). Based on Mançanares et al. (2015) and Vidane et al. (2014), the cells were trypsinized and counted through the Neubauer Chamber. In sequence the cell underwent an enzymatic reaction with trypsin (Tryple Express Enzyme (Gibco™) and centrifugation, a pellet which was resuspended with 45% DMEM (Gibco®), 10% dimethylsulfoxide (DMSO) (Sigma-Aldrich®) and 45% Fetal Bovine Serum (FBS, Hyclone, Logan, Utah, USA).

The cells were relocated in 2 cryotubes (1ml each) containing 4x10^5^ cells was transferred to Mister Frost (Nalgen®) containing isopropyl alcohol to ensure cell viability. After 24 hours the cryotubes were conditioned in liquid nitrogen for 7 days for further use. After freezing and thawing, the cells were counted with a hemocytometer using a Neubauer chamber (GridOptik/reference number - OG-500).

All the data are presented as the mean descriptive statistics analyzes.

## Results

To establish our protocol we obtained fragments of about 3 to 4 cm in size of the calamus containing the pulp and the PBS washings made the consistency of the material more malleable and consequently easier to cut lengthwise (Figure 1).

The amounts of pulp present inside the calamus varied, as did the presence of visible blood vessels. These variations were more visible among feathers of different ages, where poultrys with 12 days of age had more flesh than poultrys with 21 days and those with 34 days of age.

**Figure 2:**
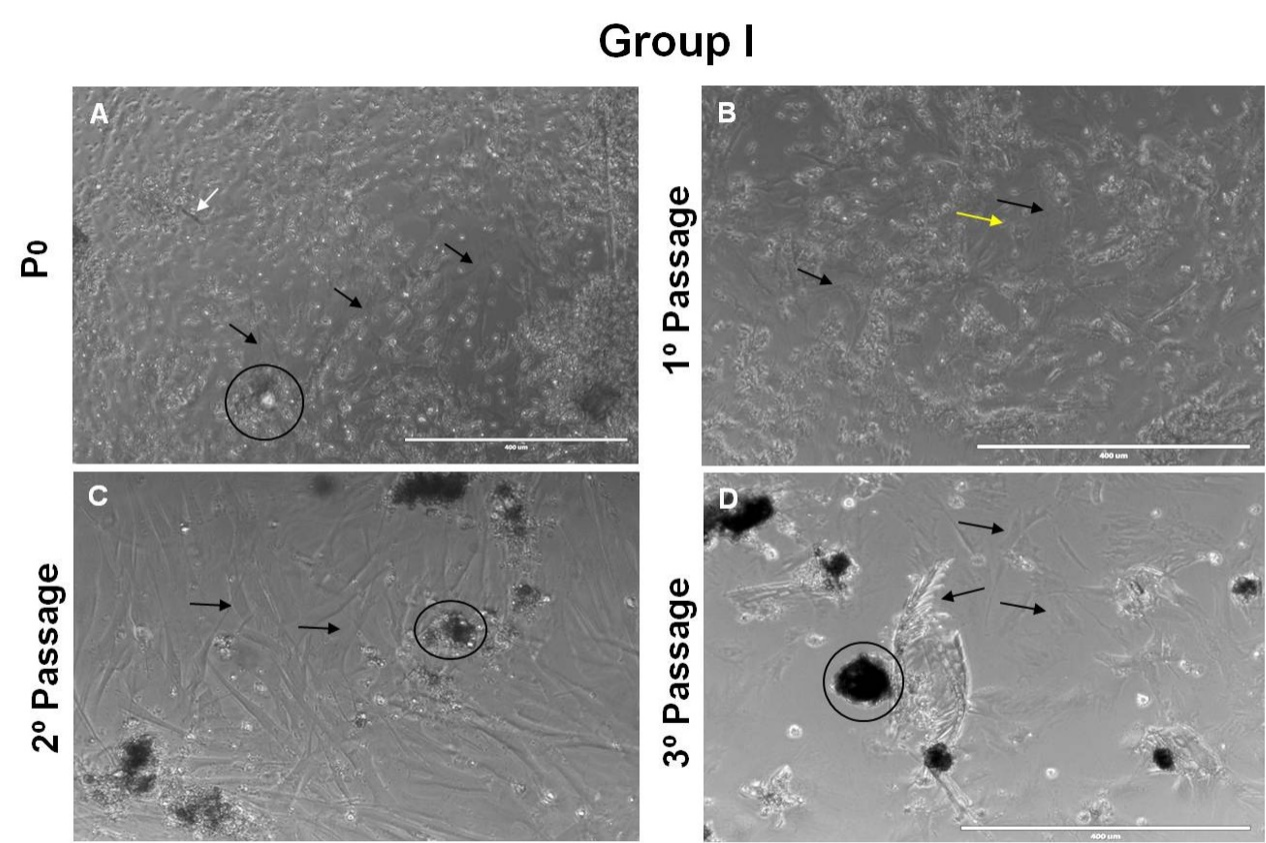
The Cell Field Overview in "P0". We observe the presence of several types of cells not adhered to the bottom of the plate, such as the appearance of cell fibers of the barbula (white arrow), besides the presence of cell clusters (circle), it is also possible to see cells adhered to the bottom of the plate with morphology similar to fibroblasts (black arrow). In B we observe a large amount of floating cells, such as erythrocytes (yellow arrow) and also a large amount of cells with fibroblast-like morphology at the bottom of the plate (black arrow), in C we observe a decrease in the amount of floating cells and an increase in the amount of cell clusters (circle) and fibroblast-shaped cells (black arrow) and in D it is possible to notice loss of the confluence, cell clusters (circle), as well as cells adhered to the bottom of the plate and barbules (black arrow). 10x magnification. Bar = 400 µm.

After 24 hours of cultivation, the primary culture was established with 80% confluence. The 1rd passage cultures were marked by a high rate of proliferation and great difference in the morphology of the cells presenting floating cells with elliptical shape and central nucleus also elliptical, rounded cells, cell clusters and cells compatible with the fibers of the barbules, in addition to cells adhered to the bottom of the plate with fibroblast format. The cell wells in 2rd already had less floating cells, but more cell clusters (Figure 2).

The subcultures were performed until the 3rd passage for 5 days and was obtained a fibroblastoid-shaped lineage cell of adhered to the plate, agglomerates cell, cells with elliptical and central nucleus shaped, round-shaped cells and similar cells to the fibers of the barbules (Figure 2). However a significant decrease of cells were present. There was presence of adhered cells but in smaller quantity than the previous passages, greater formation of cell colonies, possibly formed by keratinocytes.

Our results about cryopreservation showed which cryotube 1 present 60.6% (242.500 cells), survived and the cryotube 2, 77.6% (310.500 cells) survived. The average viability between the two cryotubes was then 68.8%, showing that approximately 70% of the cells resisted the process of dehydration and mechanical damage (Table 1).

**Table 1:** Cryopreservation of somatic cells from the follicle of bird’s.

**Somatic cells from the follicle of bird’s**	**Number of cells**	**Live cells (total)** *	**Alive (%)**	**Dead (%)**
Cryotube 1	4x10^5^	242.500	60.6%	39.4%
Cryotube 2	4x10^5^	310.500	77.6%	22.4%

* adherent cells.

Analysis of cell morphology showed that the cell present the same morphological characteristics although the number of living cells decreased visually. However the characteristics of elliptical shaped cells and central nucleus, rounded cells and fibroblast shaped cells were kept.

## Discussion

The “Biological diversity is the key to maintaining life as we know it”. The fragmentation of populations leads animals to adapt to a new environment and consequently to genetic mutation. When a population is extinguished, genes are lost that cannot be recovered (Comizzoli, 2017).

A bank of genetic resources opens up the possibility of a wide range of studies on a species ranging from genetics, toxicology, epidemiology, phylogeny or even assisted reproduction techniques (Pilot et al., 2007).

In fact, due to the lack of a bank of genetic resources of the Brazilian avian species, we have created a protocol of somatic cell culture, through a minimally invasive method, such as the cells present in the feather follicle.

Through the elaboration of this protocol we found tissue in practically all the feathers collected from the remiges, but in distinct amounts concluding that the phase of development of the feather may interfere with the amount of pulp present. Thus, feathers in the development stage present a greater amount of tissue, which goes according to Yu et al. (2004), who highlighted that in mature feathers, the pulp degenerates and its cells go into apoptosis to allow the barbs to open. This question is not related to age since the avian species have their development of feather formation in different periods compared to each other.

We obtained success in the collection and isolation method and presenting results similar to those described by Xi et al. (2003) where in their studies, after 2 hours of culture the cells from the feather follicle in primary culture were similar to fibroblasts adhered to the bottom of the plate. In our findings, after 24 hours, we obtained a confluence of cells adhered to the bottom of the plate also with a fibroblast format, demonstrating the high proliferation capacity of this type of cell.

In our study we obtained cell proliferation up to the third passage presenting a population of heterogeneous cells from elliptical shaped cells and central nucleus, rounded cells, cell clusters and cells adhered to the bottom of the plate with fibroblast shape. These results are similar to León-Quinto et al. (2009), which in order to create a cell culture of somatic cells from the skin of Lynx for the elaboration of a genetic resource bank, was able to obtain cells in culture until the third passage presenting mostly fibroblasts.

Regarding cryopreservation we obtained results above those obtained by Kjelland and Kraemer, 2012 who cryopreserved two cryotubes in his study of feather follicle cell culture and obtained fibroblast cells in both tubes, resulting in 10% viability after defrosting, due to about 90% of frozen cells did not survive cryopreservation. We obtained an average viability between the 2 cryotubes of 68.8% and maintained the morphological characteristics found in pre-freezing.

## Conclusion

Our studies prove that it is possible to initiate a culture protocol, of somatic cells from the follicle of bird’s feathers and obtain a cell line, promoting cells capable of growing and being cryopreserved. Germplasm banks are only one link within a complex chain that involves the conservation of species, so we propose an effective, viable and low invasive method as an alternative to keep avian species preserved and we encourage further studies based on this protocol, creating a biological resource bank of avian species in order to keep the various species preserved.
